# Exosomes at the crossroads of HIV-1 pathogenesis and therapeutics

**DOI:** 10.3389/fimmu.2025.1634726

**Published:** 2025-11-20

**Authors:** Pengpeng Lu, Xu Lin, Wenyi Yang, Junhao Li

**Affiliations:** 1 Beijing Institute of Hepatology, Beijing Youan Hospital, Capital Medical University, Beijing, China; 2 School of Public Health, Southern Medical University, Guangzhou, Guangdong, China; 3 The First Clinical Medicine College, Southern Medical University, Guangzhou, Guangdong, China

**Keywords:** exosomes, HIV-1 pathogenesis, viral reservoirs, co-infection, biomarkers, plant-derived nanovesicles, drug delivery, clinical translation

## Abstract

Despite advances in antiretroviral therapy (ART), human immunodeficiency virus type 1 (HIV-1) remains a global health challenge, with approximately 39 million people infected worldwide, persistent viral reservoirs, and delayed immune reconstitution. Exosomes, which are extracellular vesicles (30–150 nm) that play a key role in intercellular communication, have a dual role in HIV-1 pathogenesis and therapy. Regarding pathogenesis, this review elucidates how HIV-1 exploits the exosome pathway—hijacking the Endosomal Sorting Complex Required for Transport(ESCRT)machinery for viral budding and selectively packaging viral components, such as the accessory protein Nef, to enhance infectivity, promote immune evasion, and establish latent reservoirs. Conversely, host cells utilize exosomes to mount antiviral defense by packaging and transmitting restriction factors, such as APOBEC3G, to recipient cells. Furthermore, exosomal cargo serves as promising biomarkers for disease monitoring, and exosomes themselves are emerging as versatile therapeutic nanocarriers. We highlight that plant-derived exosomes offer unique advantages, including low immunogenicity and high scalability, for delivering next-generation antiviral agents or gene editing tools. In summary, understanding the multifaceted roles of exosomes provides crucial mechanistic insights into HIV-1 pathogenesis and unveils innovative strategies toward a functional cure.

## 1 Introduction

Since the first case of Acquired Immunodeficiency Syndrome (AIDS) was reported in 1981, human immunodeficiency virus type 1 (HIV-1) has evolved into an ongoing global public health crisis, with approximately 39 million people living with the virus worldwide in 2023, including 1.3 million new infections and 630, 000 AIDS-related deaths (1). Although antiretroviral therapy (ART) has successfully suppressed viral replication and transformed AIDS into a manageable chronic disease, significant biological and systemic challenges persist. Sub-Saharan Africa bears a disproportionate burden (2), and patients in resource-limited settings often face barriers to timely access due to socioeconomic factors (3, 4).

Despite advances in ART, including partial integrase strand transfer inhibitors (5) and long-acting regimens with improved resistance profiles (6), the path to a cure is still hampered by persistent viral reservoirs of latent proviral DNA (7), the emergence of drug resistance (8), off-target toxicity (9), and delays in immune reconstitution (10). These conditions complicate long-term adherence and predispose patients to opportunistic infections (11) and non-AIDS comorbidities (12). Achieving a functional cure, defined as sustained virological remission in the absence of ART, controlled by the host immune system, will require innovative approaches to eradicate viral reservoirs and refine treatment paradigms. Emerging strategies, such as immunomodulation through broadly neutralizing antibodies (bNAbs) (13) and CRISPR-Cas9-mediated viral genome editing (14), show preclinical promise, but still face hurdles related to variable efficacy, safety, and cost.

In this quest for a cure, extracellular vesicles (EVs), particularly exosomes, are gradually attracting academic attention. The field increasingly uses the broader term EVs, which includes exosomes, microvesicles, and apoptotic bodies. Following the Minimal Information for Studies of Extracellular Vesicles (MISEV) guidelines, this review focuses primarily on exosomes (30–150 nm), which are produced by the fusion of multivesicular bodies with the plasma membrane (15). Exosomes are enriched with tetraspanins (CD9, CD63, CD81) and heat shock proteins, and orchestrate inter-cellular communication through the transfer of biologically active transmitters (16).

Exosomes are implicated in the pathogenesis of HIV-1, demonstrating a dual role as both disease drivers and therapeutic vectors. Regarding pathogenesis, they may facilitate viral transmission through “Trojan horse-like” transport (17), suppress antiviral immunity, or deliver pathogenic factors. Conversely, exosomes also promote host antiviral responses, deliver pathogenic factors, and serve as biomarkers for early diagnosis (18). This dual role highlights their profound translational potential (19). This review is particularly timely given the recent technological breakthroughs in EV isolation and characterization, such as microfluidics and nanoparticle tracking analysis (NTA), which now allow for more precise and reproducible studies of exosome function.

## 2 Exosomes at the crossroads: pathogenesis, immune evasion, and co-infection

### 2.1 Hijacking host machinery: ESCRT-mediated viral biogenesis

**Figure 1:**
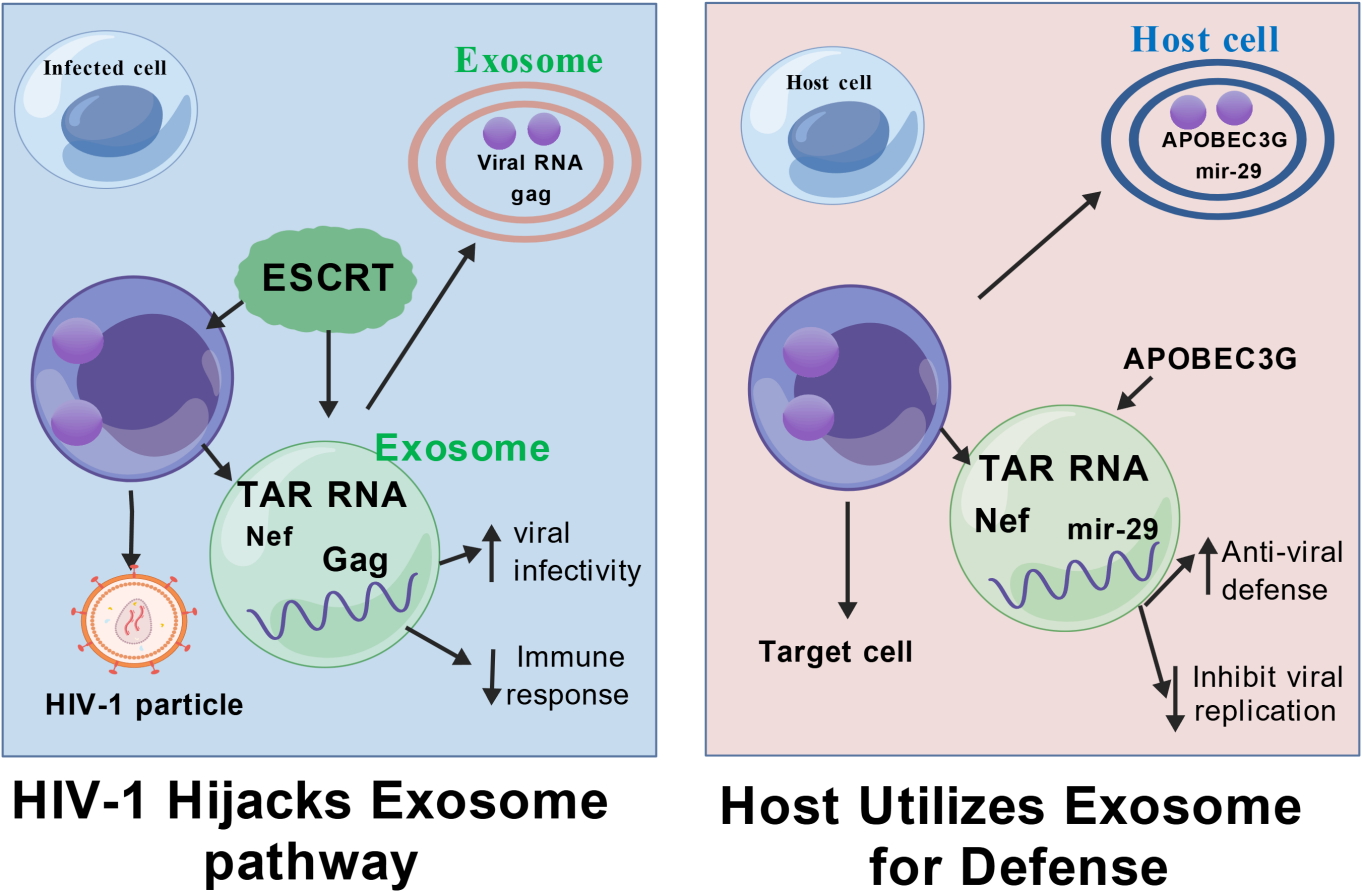
Dual role of exosomes in HIV-1 transmission and immune escape.Exosomes carry proteins, RNA, and other components that can interact with HIV-1 through the ESCRT pathway, and HIV-1 can hijack the exosome secretion pathway to deliver viral components, facilitate infection and evade immune surveillance. Exosomes are also capable of carrying viral RNA and DNA, affecting host cell gene expression and function.Figure Created with BioGDP.com.

The assembly of HIV-1 viral particles shares highly conserved molecular features with the biogenesis of exosomes, both of which rely on the sophisticated regulation of the Endosomal Sorting Complex Required for Transport (ESCRT) pathway. This mechanistic overlap suggests that HIV-1 intentionally hijacks the exosome secretory pathway as a vehicle for viral transmission and immune evasion. **Figure 1** provides a schematic overview of how exosomes are co-opted throughout the HIV-1 life cycle, highlighting the intersection of the ESCRT pathway in both viral budding and exosome biogenesis.

The HIV-1 Gag protein drives viral envelope outgrowth from host cell membranes by specifically recruiting ESCRT-I complex member TSG101 and ESCRT-III subunit CHMP4 (20, 21). This recruitment allows Gag to mimic the membrane scission process normally used to release intraluminal vesicles. Subsequently, the Vps4 protein mediates the dissociation and recycling of the ESCRT complex through hydrolysis of ATP (22), completing both viral release and the maturation of multivesicular bodies (MVBs) into exosomes (23). The resulting exosomes are often enriched with viral components (such as viral RNA and accessory proteins like Nef), facilitating their role in viral pathogenesis.

### 2.2 Dual role in immune modulation and viral transmission

During HIV-1 infection, exosomes exhibit bidirectional regulation in the microenvironment. On the one hand, viruses can deliver viral proteins and microRNAs to uninfected T cells via exosomes, significantly enhancing the susceptibility of target cells to HIV-1 by activating cellular pathways or altering surface receptor expression (24, 25). Furthermore, exosome surface molecules play a key role in viral invasion. Recent studies have demonstrated that tetraspanin family members (CD9, CD63, CD81, and CD37) can facilitate HIV-1 entry into host cells by mediating membrane fusion or endocytosis (11). Monoclonal antibodies against CD9/CD81 significantly block this exosome-dependent viral transmission, supporting the rationale for targeting these interactions.

On the other hand, host-derived exosomes can exert natural antiviral effects. They can inhibit viral replication by competitively binding to CD4+ T-cell surface receptors or by delivering restriction factors such as APOBEC3G (26–28). This finding underscores the potential for a combined therapeutic strategy targeting virus-exosome interactions: interfering with viral outgrowth through ESCRT pathway inhibitors (such as Vps4 antagonists), while concurrently modulating immune pathways and oxidative stress signaling (30). Crucially, the role of exosomes is highly dependent on their cell origin; for instance, exosomes derived from microglia may uniquely influence HIV-associated neurocognitive disorders (HAND), an area critical to addressing viral reservoir persistence.

### 2.3 Exosomes in viral co-infection and reservoir establishment

The functional complexity of exosomes is further highlighted in the context of viral and bacterial co-infection. Exosomes released in the microenvironment of HIV-1 and *Mycobacterium tuberculosis* (MTB) co-infection have been found to carry both viral RNA and pro-inflammatory factors such as TNF-α (29). These exosomes not only activate inflammatory pathways (like NF-κB), but also deliver viral components to distal cells via the paracrine pathway, promoting the establishment and reactivation of viral latent reservoirs—a crucial area for patients undergoing ART.

Coinfections with HBV or HCV also illustrate this interplay. Both HBV and HCV rely on the host ESCRT system and Rab GTPase-regulated endocytosis transport pathway for viral assembly and exosome release (31–34). Exosomes secreted by HBV-infected hepatocytes not only encapsulate intact viral particles or HBc/LHBs antigens (35), but also deliver PD-L1 proteins to induce T-cell depletion, thereby weakening the antiviral immune response and promoting latent HIV infection (36). Similarly, HCV infection-associated exosomes form transmission units with immune escape properties by carrying viral RNA-core protein complexes and host factors such as Ago2, HSP90, and miR-122 (37). While miR-122 enhances viral replication by stabilising HCV RNA, a related pathway involves exosome-mediated activation of the miR-19a/SOCS3/STAT3/TGF-β signaling axis, which synergistically promotes hepatic fibrosis progression (38, 39). Specifically, miR-19a targets SOCS3, which in turn de-represses the STAT3 pathway, leading to increased expression of the profibrotic cytokine TGF-β. It is therefore suggested to establish a dynamic monitoring and joint intervention system for co-infection, aiming to break through existing treatment bottlenecks and achieve functional cure of HIV-1 and effective control of co-infection complications through interdisciplinary cooperation and preclinical model optimization.

### 2.4 Exosomal cargo specificity and heterogeneity from diverse reservoirs

Beyond T cells, HIV-1 infects a broad spectrum of cells, including macrophages, microglia, and dendritic cells, each contributing uniquely to viral reservoir persistence and the clinical challenges of ART-mediated remission. Exosomes derived from these diverse cellular reservoirs exhibit distinct compositions and functional properties. For instance, microglia-derived exosomes have been centrally implicated in HIV-1-associated neurocognitive disorders (HAND), carrying neurotoxic viral proteins (such as Nef or Tat) and inflammatory cytokines that disrupt neuronal function (17). Similarly, macrophages, which are key tissue reservoirs of latent HIV-1, release exosomes that are often enriched with pro-inflammatory microRNAs (miRNAs) and viral components. These exosomes can facilitate viral dissemination and significantly modulate the immune microenvironment in distant tissues (40).

Crucially, the process by which these viral components are incorporated into exosomes is not merely passive but is governed by highly selective packaging mechanisms. This targeted inclusion ensures the delivery of pathogenic molecules essential for viral spread and persistence. For example, both HIV-1 proteins (such as Nef and Gag) and viral RNAs (like TAR) are highly enriched in exosomes from infected cells (41, 42). Nef, a potent accessory protein, modulates exosome biogenesis and content, promoting the secretion of vesicles that enhance infectivity and impair immune responses in recipient cells (43). Similarly, the TAR RNA element—a key non-coding viral component—is abundantly packaged into exosomes and can manipulate gene expression in target cells to foster a proviral environment (44).

Understanding these specific compositions and packaging rules across different cell types is crucial for developing comprehensive “shock and kill” or “block and lock” strategies to eliminate the persistent HIV-1 reservoir and effectively treat its major comorbidities.

## 3 Exosomes as biomarkers for HIV-1 diagnosis and disease monitoring

### 3.1 Clinical rationale and diagnostic potential

**Figure 2:**
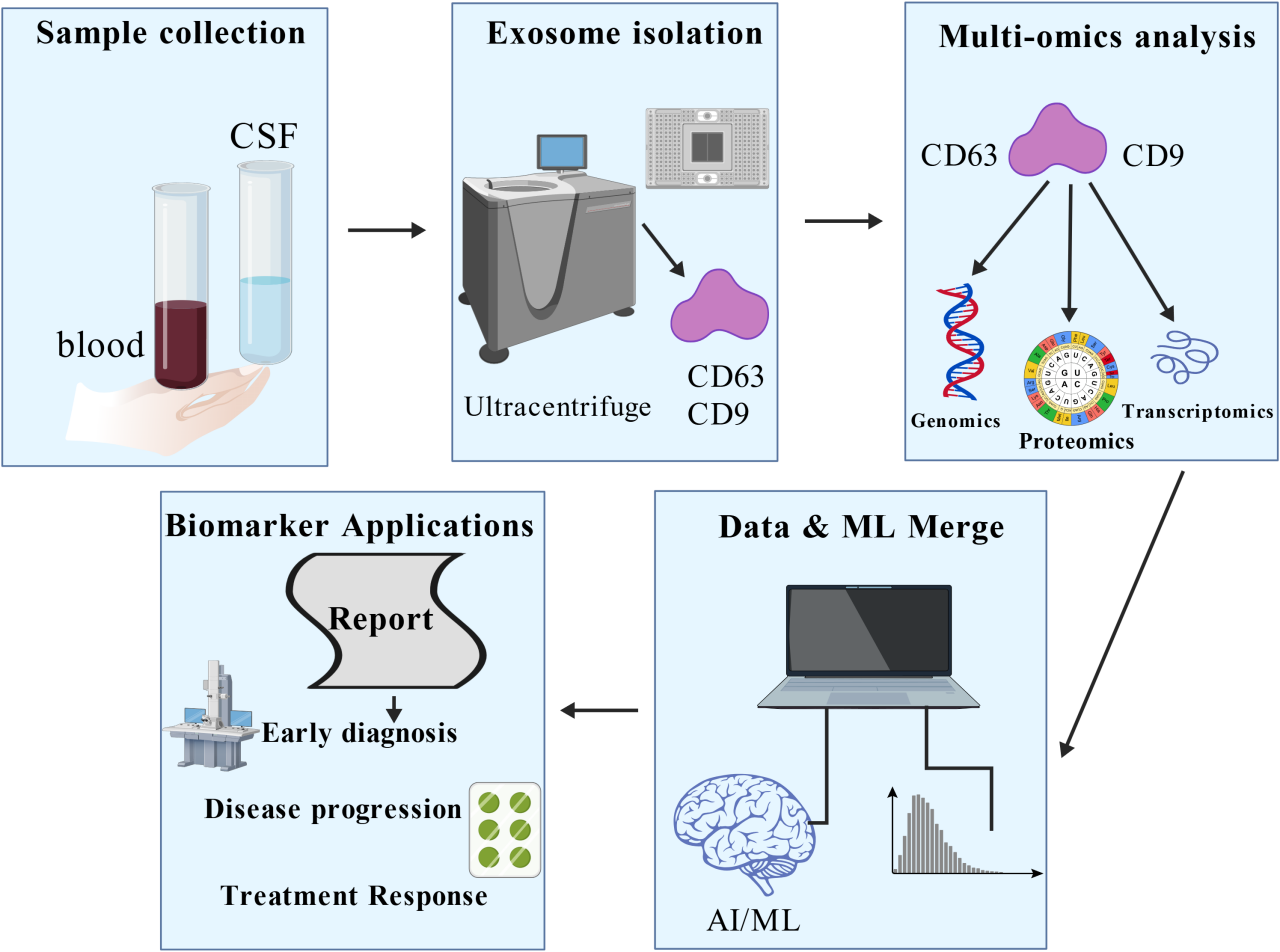
Potential applications of exosomes in the diagnosis and treatment of HIV-1 infection. Exosomes can be extracted through human samples. Exosomes carry a variety of components, including proteins and RNA, which can be used as biomarkers for detecting viral load. The molecular characteristics of these exosomes are analysed in depth using advanced analytical techniques such as machine learning to help assess disease progression and immune reconstitution. Figure Created with BioGDP.com.

Exosomes have become novel candidate markers for disease diagnosis and progression monitoring due to their unique biological properties (45). The nucleic acids, proteins, and lipid components they carry not only dynamically reflect the pathophysiological state of host cells (46–49), but also provide an ideal sample source for non-invasive liquid biopsy due to the protective effect of the exosome membrane structure, which makes its contents highly stable in body fluids. **Figure 2** illustrates the translational potential of exosomal biomarkers across different stages of HIV-1 infection, from early diagnosis to reservoir detection. This stability and ability to reflect tissue-specific status give them a distinct advantage over traditional plasma markers. Some studies have shown that in fields such as neurodegenerative diseases and renal fibrosis, the aberrant expression of exosome-specific miRNAs (such as miR-21 and the miR-29 family) opens new avenues for early diagnosis and assessment of therapeutic efficacy (50, 51). In conclusion, the diagnostic value of exosomes in HIV-1 infection is gradually being highlighted, with a specific focus on identifying signatures that correlate with chronic inflammation and immune dysregulation.

### 3.2 Molecular signatures and AI-driven discovery for disease monitoring

Studies have shown that the number of exosomes in the plasma of HIV-infected patients without antiretroviral therapy (ART) is significantly increased, and their particle size distribution is shifted to a larger size. The molecular cargo contained within these exosomes provides robust indicators of disease progression, characterized by up-regulated miR-155 and miR-146a and abnormal elevation of oxidative stress markers such as 8-OHdG and MDA (52). A cohort study further indicated that plasma exosome abundance was significantly and positively correlated with absolute CD8+ T-cell counts (53), a correlation that provides a potential biological basis for assessing the state of immune reconstitution and systemic inflammation in HIV-infected patients undergoing ART.

Despite the richness of molecular information contained in exosomes (54), screening for specific markers that are highly correlated with HIV-1 infection progression remains challenging. Traditional biomarker discovery strategies are limited by the complexity of multidimensional data. The introduction of machine learning techniques has provided a breakthrough direction for this bottleneck. It has been proposed that random forest classifiers can significantly outperform the predictive efficacy of traditional statistical methods by integrating exosomal proteomics, transcriptomics, and clinical parameters in the application scenario of identifying cancer-related markers (55, 56). This methodological advancement drives innovation for HIV research: an integrated analysis framework based on deep learning-driven fusion of multi-omics data and artificial intelligence may resolve key feature profiles associated with viral load, latent reservoir activation, or immune exhaustion from the exosomal molecular network, leading to accurate disease stratification models (57). Future studies need to further validate the applicability of such computational biology strategies in longitudinal HIV cohorts to accelerate the clinical translation of exosome markers.

## 4 Exosomes as nanocarriers for anti-HIV-1 therapy: a comparative perspective

### 4.1 Unique advantages and targeted delivery of plant exosomes

**Figure 3:**
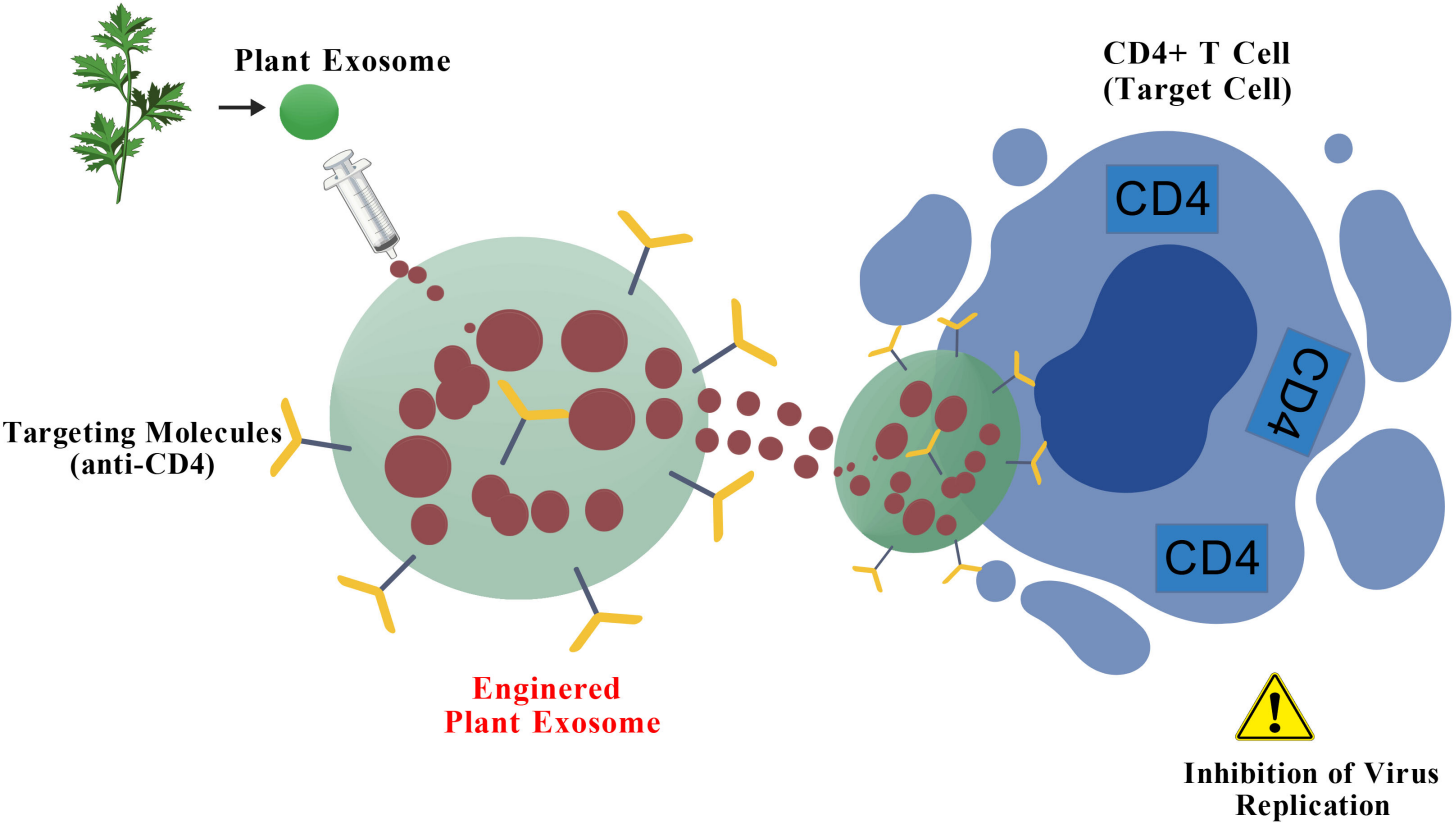
Potential of plant-derived exosomes for HIV-1 therapy. After exosomes are extracted from plants, they can be engineered to carry chemical drugs. These modified exosomes can be used as drug delivery systems to target anti-HIV drugs to target cells such as CD4+ T cells, thereby increasing therapeutic efficacy and reducing the impact on non-target cells. This process combines the natural advantages of plant-derived exosomes with modern bioengineering and drug design techniques, providing innovative ideas for developing novel anti-HIV therapeutic strategies. Figure Created with BioGDP.com.

Plant-derived exosomes (also often termed exosome-like nanoparticles or HELNs) have emerged as promising nanocarriers for antiviral therapy, offering unique advantages such as high biocompatibility, inherent stability, and the demonstrated ability to cross biological barriers like the blood-brain barrier (BBB) (58–66). Their structural similarity to mammalian exosomes, coupled with the abundance of bioactive molecules they carry, makes them an attractive, scalable platform for therapeutic agent delivery. **Figure 3** summarizes this potential: after extraction from plants, these exosomes can be engineered to carry anti-HIV chemical drugs. These modified exosomes serve as targeted drug delivery systems, aiming for specific cells like CD4+ T cells, thereby increasing therapeutic efficacy and minimizing off-target toxicity. This process effectively combines the natural advantages of plant-derived exosomes with modern bioengineering and drug design techniques. Studies have highlighted their potential for targeting HIV-1 related pathology. For instance, grapefruit-derived exosomes exhibit high drug-loading efficiency and protective properties (58). Similarly, ginger exosomes have shown potential in modulating aberrant immune responses and reducing inflammatory pathways (63), which could be beneficial in managing the chronic immune activation and non-AIDS comorbidities associated with HIV-1 infection. Their proven capacity for CNS penetration further addresses a critical challenge in HIV-1 treatment, offering an ideal carrier platform for eradicating viral reservoirs in the brain (66).

### 4.2 Comparative analysis and translational challenges

While plant exosomes present a promising, cost-effective alternative to mammalian vectors—particularly for their high scalability and generally low immunogenicity—a clearer perspective requires a critical comparison against alternative delivery systems, as summarized in **Table 1**. However, the translation of plant exosomes into clinical use is not without significant critical challenges. Variability in molecular cargo and quantity between plant sources, potential host-specific immunogenicity, and a limited understanding of their precise mechanistic behavior in human systemic circulation remain major hurdles (58, 74, 78). In conclusion, to fully realize their potential in HIV-1 therapy, future research must address three crucial bottlenecks: standardization of isolation protocols (to ensure consistency and homogeneity), rigorous safety and pharmacokinetic profiling *in vivo*, and clarification of the complex global regulatory pathways (72–80). This concerted effort is necessary to move these highly promising natural nanocarriers from the bench to the bedside.

**Table 1:** Comparison of plant-derived and mammalian-derived exosomes as drug delivery vehicles.

Feature	Plant-derived exosomes	Mammalian-derived exosomes	Reference
Source	Wide variety of plants (e.g., grapefruit, ginger)	Cell cultures (e.g., HEK293, MSCs) or bodily fluids	(15, 58, 66)
Immunogenicity	Generally low, but potential for plant-specific antigens	Higher risk of immune rejection, especially allogeneic	(58, 78)
Scalability	Highly scalable; cost-effective agricultural production	Complex and expensive cell culture processes	(58, 74)
Loading Method	Often passive loading or pre-loading via plant cultivation	Often requires active loading (electroporation, transfection)	(19, 60)
BBB Penetration	Demonstrated ability for many types	Variable; often requires engineering	(66)
Regulatory Path	Less defined; may be classified as dietary supplements	Better defined but complex (ATMPs in EU, biologics in US)	(74, 79, 80)

BBB, blood-brain barrier; MSCs, mesenchymal stem cells; ATMPs, Advanced Therapy Medicinal Products.

## 5 Outlook and future directions: addressing translational hurdles

The clinical translation of exosomes in HIV-1 therapy faces multidimensional challenges, demanding systematic breakthroughs that reconcile their dual biological attributes and therapeutic potential.

### 5.1 Challenges in clinical trial design and quality control

The clinical translation of exosomes in HIV-1 therapy is fundamentally constrained by deep technical and biological contradictions. First, the primary obstacle in clinical trial design stems from exosome biological heterogeneity. The dual roles of exosomes as both viral transmission vectors and potential therapeutic tools (67–70) challenge traditional efficacy assessment systems centered on viral load (71). Differences arising from host factors and preparation processes further complicate single-cohort studies, with inconsistencies in particle detection methods introducing data bias (74). This urgently mandates the establishment of a functionally validated quality control system, standardizing key parameters like target delivery efficiency and pathogen clearance (72, 73, 75). Furthermore, novel joint endpoint indicators, such as latent reservoir-specific miRNA profiles combined with single-cell sequencing, are required to resolve the exosomal dynamic reprogramming of the immune microenvironment (76, 77).

Second, safety and scale-up pose significant hurdles. Despite the low immunogenicity of natural exosomes, risks persist: exosomes from HIV-infected cells may carry viral proteins like gp120, necessitating stringent pathogen clearance processes (78). Large-scale production of engineered exosomes is challenged by batch-to-batch heterogeneity, where fluctuations in loading efficiency compromise therapeutic consistency (75). Ultimately, systematic assessment of long-term exposure risks in preclinical models is required, especially regarding the dynamic interplay between exosomes and vulnerable microenvironments such as the Central Nervous System.

### 5.2 Regulatory barriers and future roadmap: integrating an interdisciplinary ecosystem

The ambiguity of the regulatory framework severely exacerbates the translational dilemma. The transboundary properties of exosomes lead to classification controversies: the EU classifies functional RNA-carrying exosomes as Advanced Therapeutic Medicinal Products (ATMPs) (79), while the US FDA dynamically adjusts classification criteria based on functional properties (80). This regulatory uncertainty directly leads to a fragmented quality control system, evidenced by the lack of international consensus on assays for miRNA quantification or CRISPR complex activity (81, 82). Although countries like Japan and South Korea have attempted to standardize production processes through special guidelines (83, 84), there is still an urgent need to establish a cross-regional adaptive regulatory pathway focused on resolving the ethical review and biosafety assessment challenges of exosomes carrying gene editing tools (79, 80). Furthermore, achieving a paradigm breakthrough in HIV-1 therapy requires an interdisciplinary synergistic system spanning virology, nanomedicine, and policy sciences. This system involves action at three levels: focusing research on exosomes as intelligent carriers at the “virus-host interaction interface” (Mechanism Level); promoting the process integration of technologies like ultracentrifugation and microfluidics to establish end-to-end quality control standards (Translational Strategy); and leveraging ATMP management experience to formulate a function-oriented dynamic classification strategy (Regulatory Adaptation). Only through the triple drive of technology iteration, standardization, and policy innovation can exosomes move from a laboratory concept to a strategic tool for clinical antiretroviral therapy.

## 6 Conclusion and future outlook

This review has elucidated the dual role of exosomes in HIV-1 pathogenesis, highlighting their critical capacity to facilitate viral spread (via ESCRT-mediated packaging and reservoir-specific cargo) while simultaneously serving as promising therapeutic and diagnostic tools. Translating this potential from bench to bedside necessitates overcoming significant, interlinked hurdles.

A primary challenge lies in the inherent heterogeneity and lack of standardization in exosome isolation and characterization. Current methods yield preparations with varying purity and composition, which complicates data interpretation and demands the development of robust, scalable, and reproducible manufacturing processes (73). Furthermore, the biological complexity requires a deeper understanding of their *in vivo* fate, targeting specificity, and potential off-target effects, particularly when engineered as drug delivery vehicles (19). The regulatory landscape remains ambiguous; clarifying whether these products are classified as drugs, biologics, or advanced therapies is essential for defining a clear path to clinical approval (79). Ultimately, future research must bridge knowledge gaps—such as the precise mechanisms of exosome-mediated reservoir establishment and reactivation—by harnessing multidisciplinary approaches like synthetic biology and machine learning to design smart exosomes capable of targeted reservoir elimination and immune reconstitution.

By addressing these challenges through concerted efforts in basic science, manufacturing engineering, and regulatory collaboration, exosome-based strategies may eventually evolve into cornerstone technologies for achieving a functional cure for HIV-1. (All figures in this paper via BioGDP (85)).
